# Long-range vortex transfer in superconducting nanowires

**DOI:** 10.1038/s41598-019-48887-7

**Published:** 2019-08-27

**Authors:** Rosa Córdoba, Pablo Orús, Željko L. Jelić, Javier Sesé, Manuel Ricardo Ibarra, Isabel Guillamón, Sebastián Vieira, Juan José Palacios, Hermann Suderow, Milorad V. Milosević, José María De Teresa

**Affiliations:** 1 Instituto de Ciencia de Materiales de Aragón (ICMA), Universidad de Zaragoza-CSIC, E-50009 Zaragoza, Spain; 2 Departamento de Física de la Materia Condensada, Universidad de Zaragoza, E-50009 Zaragoza, Spain; 3 University of Antwerp, Department Physics, Groenenborgerlaan 171, B-2020 Antwerp, Belgium; 4 Laboratorio de Microscopías Avanzadas (LMA)-Instituto de Nanociencia de Aragón (INA), Universidad de Zaragoza, E-50018 Zaragoza, Spain; 5 Laboratorio de Bajas Temperaturas, Departamento de Física de la Materia Condensada, Instituto de Ciencia de Materiales Nicolás Cabrera, Condensed Matter Physics Center (IFIMAC), Universidad Autónoma de Madrid, 28049 Madrid, Spain; 6 Departamento de Física de la Materia Condensada, Condensed Matter Physics Center (IFIMAC), Universidad Autónoma de Madrid, 28049 Madrid, Spain; 7 Present Address: Instituto de Ciencia Molecular, Universitat de València, Catedrático José Beltrán 2, Paterna, 46980 Spain

**Keywords:** Electronic properties and materials, Nanowires, Superconducting properties and materials

## Abstract

Under high-enough values of perpendicularly-applied magnetic field and current, a type-II superconductor presents a finite resistance caused by the vortex motion driven by the Lorentz force. To recover the dissipation-free conduction state, strategies for minimizing vortex motion have been intensely studied in the last decades. However, the non-local vortex motion, arising in areas depleted of current, has been scarcely investigated despite its potential application for logic devices. Here, we propose a route to transfer vortices carried by non-local motion through long distances (up to 10 micrometers) in 50 nm-wide superconducting WC nanowires grown by Ga^+^ Focused Ion Beam Induced Deposition. A giant non-local electrical resistance of 36 Ω has been measured at 2 K in 3 μm-long nanowires, which is 40 times higher than signals reported for wider wires of other superconductors. This giant effect is accounted for by the existence of a strong edge confinement potential that hampers transversal vortex displacements, allowing the long-range coherent displacement of a single vortex row along the superconducting channel. Experimental results are in good agreement with numerical simulations of vortex dynamics based on the time-dependent Ginzburg-Landau equations. Our results pave the way for future developments on information technologies built upon single vortex manipulation in nano-superconductors.

## Introduction

Vortices in type-II superconductors can be driven out of equilibrium by the joint action of an external magnetic field and an applied electrical current. The motion of the vortices hampers the dissipation-free transport of the electrical current, requiring suitable strategies to achieve vortex immobilization^1–5^. During the last decades, great attention has been given to the investigation of the vortex dynamics arising from local driving forces occurring where the current flows (the so-called *local* geometries). These phenomena have been exploited for the realization of rectifiers^6^ and vortex diodes^7–9^, and for the enhancement of superconducting critical parameters^10^. Moreover, at the mesoscopic level, the behavior of the vortices in one and two-dimensional superconducting systems (nanowires and ultra-thin films, respectively) can be drastically influenced by spatiotemporal dependent potentials, such as geometric confinement^11–14^, and spatial^15^ and temporal pinning^16^. The control of vortices in the local geometry is of paramount importance for the development of superconducting logic gates^7^, quantum switches^17^, and single-photon detectors^18–20^.

On the other hand, *non-local effects* related to motion of vortices in regions of superconductors depleted of current have also been reported. Such systems are crucial for studying the phenomena of charge imbalance^21^ and electron entanglement through crossed Andreev reflection^22,23^. Regarding vortex flow, non-local phenomena due to vortex-vortex interaction have been reported in Corbino disks^24^, while the most notable example is Giaever’s flux transformer^25^ realized in junctions made of layered superconductors^26^, where vortices presented electromagnetic coupling across an insulating barrier. More recently, non-local vortex motion in Hall-bar-shaped superconducting nanowires has been reported^27–31^. In such configuration, the vortex-vortex interaction enables the transfer of momentum amongst the vortices along the nanowire over large distances (3 μm).

**Figure 1:**
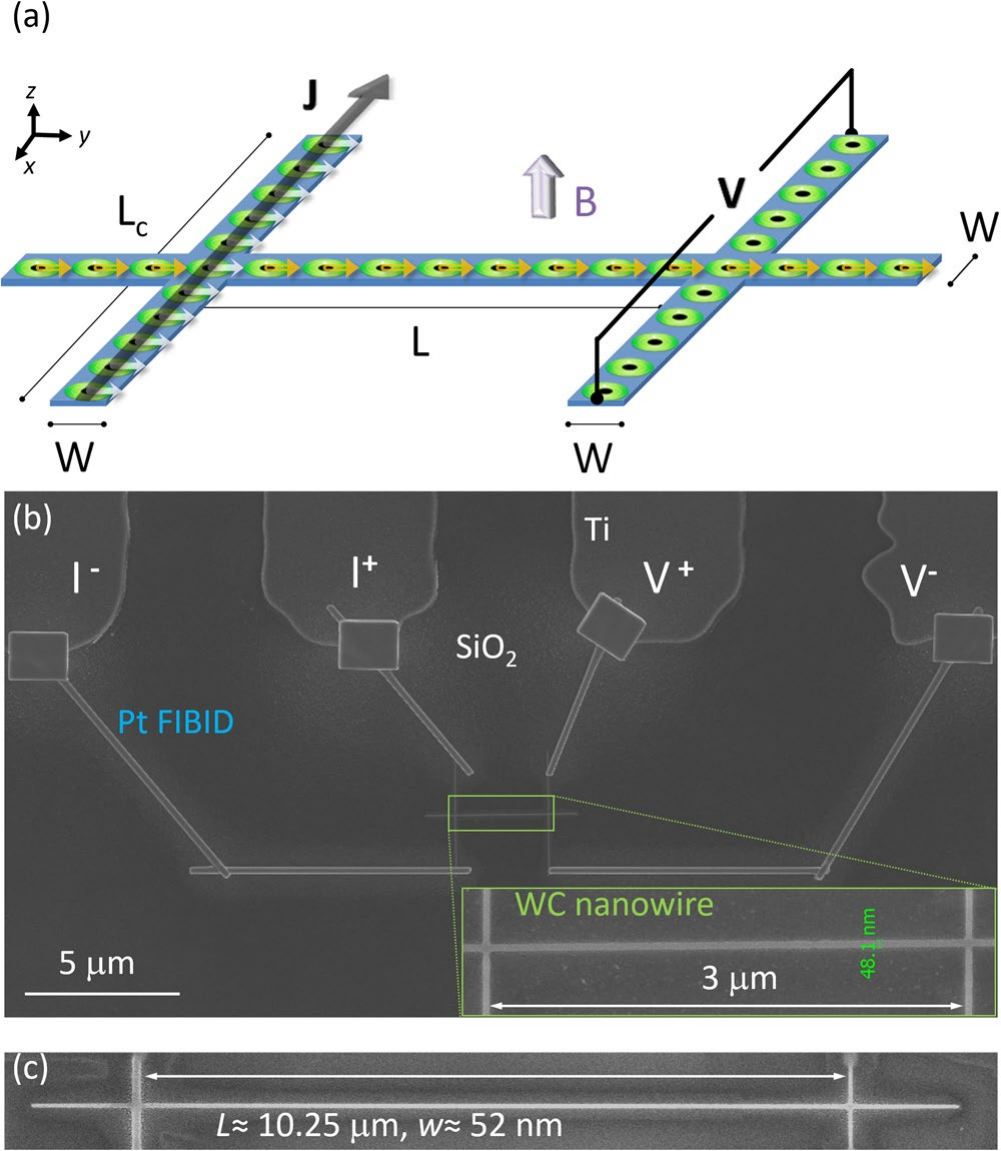
(**a**) Overview of the sample geometry. Superconducting nanowire of width *W* and length *L*, with current and voltage contacts of length *L*_*c*_ and width *W*. Injection of the current density *J* occurs at the contacts on the left side, while contacts on the right side of the device are used to measure the non-local voltage *V*. Non-local transport of superconducting vortices occurs along the length of nanowire. Vortices are indicated by green/black circles. The Lorentz force being exerted on the vortices is indicated by white arrows. Non-local vortex motion is indicated by yellow arrows. (**b**) SEM image of the WC nanostructure (nanostructure A-short) for non-local electrical measurements. Inset shows a high magnification SEM image of the nanostructure A-short. (**c**) SEM image of the nanostructure A-long.

The geometry employed to detect non-local vortex motion in such systems consists of a superconducting nanowire of length *L*, width *W*, and thickness, with two transversal leads of length *L*_*C*_ and width *W* perpendicularly crossing at its ends (Fig. 1(a)). The bias current flows through one transversal lead and the voltage is detected in the other^27^. Vortices in the current lead and the closest few vortices in the longitudinal nanowire experience a push from the local force, perpendicular to the directions of the applied magnetic field and the bias current. The rest of vortices in the longitudinal section of the nanowire feel the transfer of momentum via the vortex-vortex interaction. If this motion can be sustained up to the location of the voltage lead, the electric field associated with a moving vortex crossing the lead will generate a potential difference, yielding the non-local voltage. The non-local voltage and non-local resistance (calculated as the ratio of the non-local voltage and the bias current) have been found until now to be at most ~0.8 μV and ~1 Ω^29^, respectively. The enhancement of such output values is key for building effective superconducting electronic devices, requiring less signal amplification.

In this work, we report the long-range vortex transfer carried in 50 nm-wide superconducting WC nanowires grown by Ga^+^ Focused Ion Beam Induced Deposition (FIBID)^32,33^. A giant non-local electrical signal is detected far away from the bias current leads, at large distances (3 and 10 μm) compared to the intervortex distance (a few tens of nanometers). We have found this giant signal in a wide temperature range (0.1T_c_–0.7T_c_). Furthermore, the signal in 50 nm-wide nanowires is nearly two orders of magnitude higher than for the 200 nm-wide ones. This huge enhancement can be attributed to the geometric confinement of a single vortex row at the center of the nanowire that prevents transversal vortex displacements^11,13,34^. Such a long-range coherent vortex displacement along microns can be explained by the open boundary conditions at the outer ends of the channel produced by the specific nanofabrication technique. The experimental data are supported by numerical simulations performed within the time-dependent Ginzburg-Landau (TDGL) framework.

## Results and Discussion

We focus first on results obtained in 50 nm-wide and 3 µm-long structures, for which only a single row of vortices can be accommodated in the nanowire. In addition, we have also studied longer and wider structures to investigate the effects of long-range transfer and several vortex rows fitting in. Specifically, the dimensions of 50 nm-wide samples are: 50 nm in width, 20 nm in thickness and 3 μm in length (sample A-short hereafter, inset of Fig. 1(b)); and 52 nm in width, 40 nm in thickness and 10.25 µm in length (sample A-long hereafter, Fig. 1(c)). The dimensions of wider samples (type B hereafter) are: 200 nm in width, 200 nm in thickness and 3 μm in length. Further experimental details, including microstructure and composition obtained by Transmission Electron Microscopy, are described in the Supporting information (Fig. S1).

We use a typical four-probe local configuration for the magnetotransport measurements (see Supporting information, Fig. S2 and Fig. S3). The local resistance of the sample A-short at the normal state (10 K) R_N_ is 6563 Ω and its resistivity ρ_N_ equals 219 μΩcm, in agreement with previous values reported in the literature^11,33^. At low temperature, the nanowire shows a sharp resistance drop at T_c_ ≈ 4.47 K (defined as the temperature at which the resistance value equals 0.5R_N_), entering the superconducting state. A reentrance of the superconductivity induced by high magnetic fields (~1.85 T) is also observed in these samples, due to the geometric confinement of a single row of vortices formed at the center of the nanowire while superconductivity is established at its edges^11,34^. For WC wires with lateral size broader than ~ 70 nm, more vortex rows can fit into the wire preventing this phenomenon, although resistance oscillations related to the entrance of an increasing number of vortex rows into submicron wires of other materials have been reported^35–37^.

Vortex motion within the nanowire is restricted by the existence of potential barriers along all edges of the nanostructure, which hamper transversal vortex displacements and prevent vortices from leaving the nanowire. For a given value of applied magnetic field B, when the value of the driving current density J reaches a critical value J_c_(B), the Lorentz force it exerts is strong enough to make the vortices overcome these barrier walls and transversally travel along the nanowire. Additionally, the intrinsic pinning of the material introduces a threshold for the Lorentz force that must be overcome for the motion to start, which is achieved at a certain value of the driving current density, J*(B), below J_c_(B). The onset of vortex motion is facilitated as pinning sites get saturated, resulting in a reduction in the value of J*(B) when the magnetic field, and thus the vortex density, is increased.

The non-local sample geometry is depicted in Fig. 1. When the value of J*(B) is reached, vortex motion starts at the crossing point between the current lead and the longitudinal section of the nanowire. Momentum is transferred to other vortices in the longitudinal section of the nanowire via the vortex-vortex interaction and is supported by edge confinement along that section. Passing vortices in the second crossing point at the voltage lead yield the non-local voltage.

**Figure 2:**
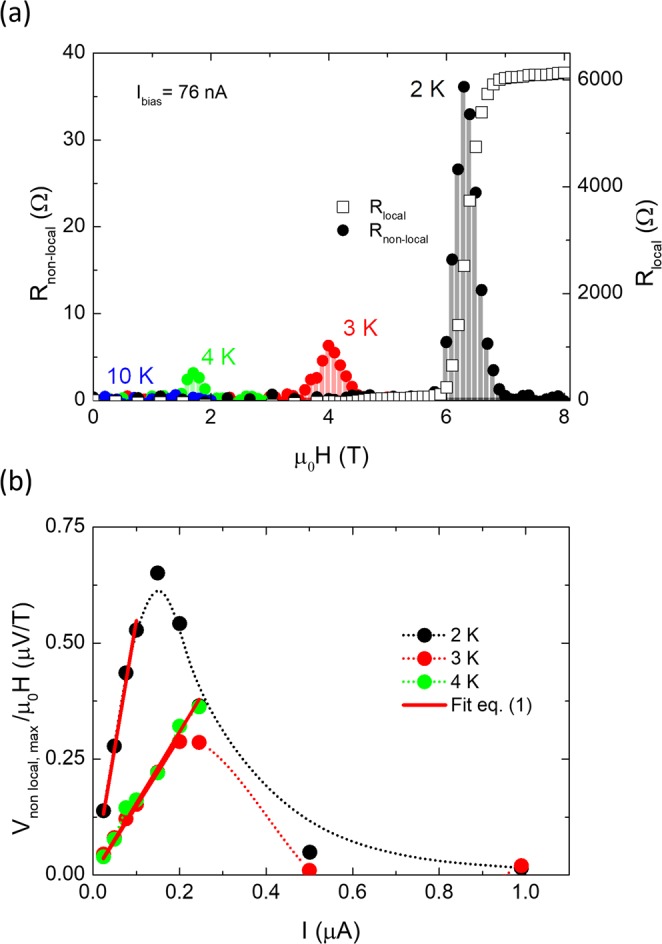
(**a**) Magnetic field dependence (at 2 K, 3 K, 4 K and 10 K) of the non-local resistance using a bias current of 76 nA (left *y*-axis) and local resistance at 2 K using the same current (right *y*-axis) for the nanostructure A-short. (**b**) Normalized maximum non-local voltage as a function of the current at 2 K, 3 K and 4 K for the nanostructure A-short. The dotted lines are a guide to the eye.

The non-local electrical signal of the nanostructure A-short was studied at several temperatures, 2 K (0.45T_c_), 3 K (0.67T_c_), 4 K (0.89T_c_) and above the superconducting state, 10 K, as a function of magnetic field, up to 9 T, perpendicularly applied to the substrate (Fig. 2(a)). The bias current density flowing through the left lead had a value of 0.076 MAcm^−2^ (I_bias_ = 76 nA), located below the zero-field critical current density J_c_(B = *0)* at those temperatures, J_c_ (2 K) = 0.24 MAcm^−2^, J_c_ (3 K) = 0.18 MAcm^−2^, J_c_ (4 K) = 0.09 MAcm^−2^. We found a zero R_non-local_ value at 2 K below μ_0_H~5.7 T. A linear dependence of R_non-local_ with μ_0_H was detected from ~5.7 T to ~6.3 T; the latter being the field at which the maximum value of R_non-local_, 36 Ω, was achieved. Above μ_0_H~6.3 T, R_non-local_ decreased until μ_0_H~7.1 T (above B_c2_(0.9R_N_) = 6.8 T), a field value at which the nanowire reached the normal state and a zero R_non-local_ value was recovered. This huge R_non-local_ value is more than 40 times higher than those reported in wires with lateral size from 70 nm to 2 μm^29^. The maximum R_non-local_ and the corresponding magnetic field value both decrease with temperature in the superconducting state (from 2 K to 4 K), vanishing above T_c,_ as it occurred at 10 K.

**Figure 3:**
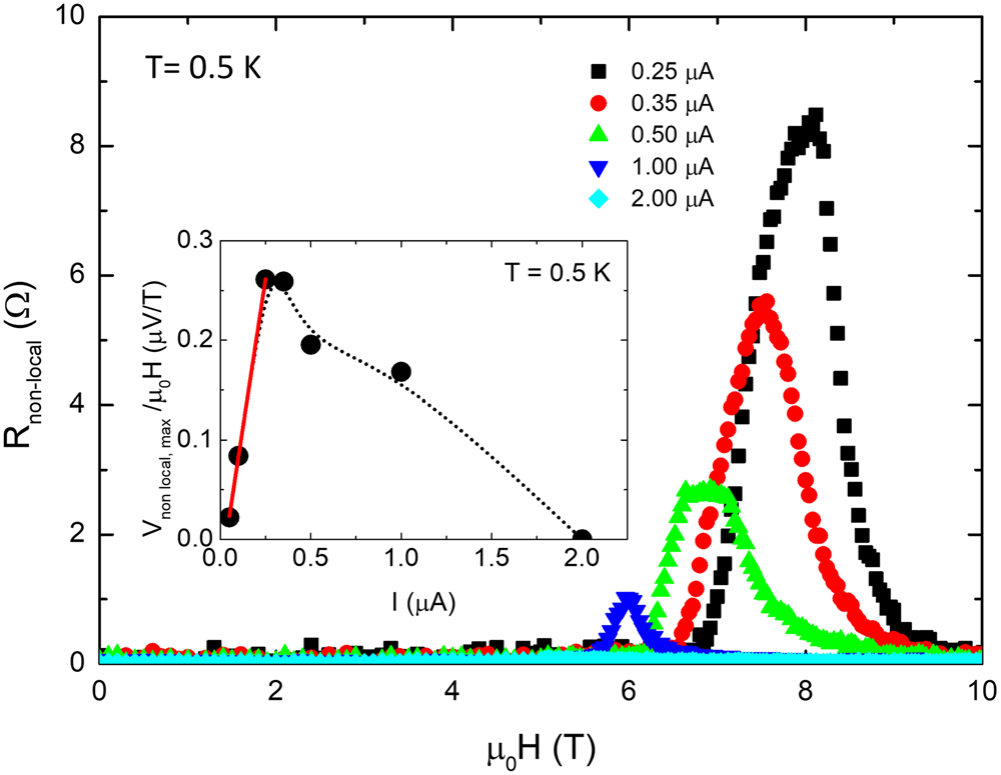
Magnetic field dependence of the non-local resistance at 0.5 K using bias currents from 0.25 μA to 2 μA for the nanostructure A-long. Inset shows normalized maximum non-local voltage as a function of the current at 0.5 K. The red line is the fit to Eq. (1) and the dotted line is a guide to the eye.

The same R_non-local_ dependence with μ_0_H was detected for both nanostructure A-long (Fig. 3) and nanostructure B (Fig. S4 in the Supporting information). In the former, R_non-local,max._ was found to be 9 Ω at 0.5 K (0.1T_c_) and within a magnetic field range of b = B/B_c2_ = [0.50–0.87], in the latter, R_non-local_,_max._ reached 0.15 Ω at 2 K (0.4T_c_).

Helzel^29^ *et al*. proposed a model to explain the linear dependence of the measured non-local electrical signal with the electrical current. Taking into account the sample geometry, and the vortex density n_ϕ_ = µ_0_H/ϕ_0_, there is a number of vortices N = n_ϕ_W^2^ at the crossing point which are affected by the Lorentz force F_L_ = Jϕ_0_t due to the applied current density J = I/Wt. These vortices exert pressure onto the neighboring ones located in the longitudinal wire, with a magnitude given by $p=\frac{{n}_{{\rm{\Phi }}}{{\rm{\Phi }}}_{0}I}{t}$. The resultant force on the vortices in the longitudinal nanowire per unit length is F = pW, balanced by the frictional force to move vortices in a viscous medium ${F}_{f}={n}_{{\rm{\Phi }}}LW\eta {v}_{\phi }$ where η and v_φ_ are the viscosity and the vortex velocity in the NW, respectively. Defining ${V}_{non-local}=W{\mu }_{0}H{v}_{\phi }$, one obtains:

1 $${V}_{non-local}=\frac{W{\mu }_{0}{H{\rm{\Phi }}}_{0}I}{\eta Lt}$$

2 $${R}_{non-local}=\frac{{V}_{nonlocal}}{I}=\frac{W{\mu }_{0}{H{\rm{\Phi }}}_{0}}{\eta Lt}$$

From the R_non-local_ versus μ_0_H curves of Fig. 2(a) taken at different bias currents, we plot the maximum V_non-local_ values, normalized by the applied magnetic field, as a function of the applied current (Fig. 2(b)). Using the model defined in Eq. (1), one can fit the linear current dependence of the V_non-local, max_/μ_0_H. It is remarkable that the linear dependence with the current starts from very low current values, indicating that in this field range, intrinsic pinning plays a minor role and the effect is governed by vortex viscosity^38^. The linear dependence exists up to J_c_(B), beyond which V_non-local, max_/μ_0_H starts to decrease.

The V_non-local_/μ_0_H decay with temperature is plausibly due to the reduction of the confinement potential and the increase of the thermal energy, which decreases the vortex lattice rigidity^27^ and finally leads to vortex line melting^36,39^. A similar dependence with temperature has also been observed in nanostructure B (Fig. S5(a)), in which non-local resistance was detected within the temperature range t = T/T_c_ = [0.1–0.6] and magnetic field range b = B/B_c2_ = [0.40–0.96] (Fig. S5(b)).

**Figure 4:**
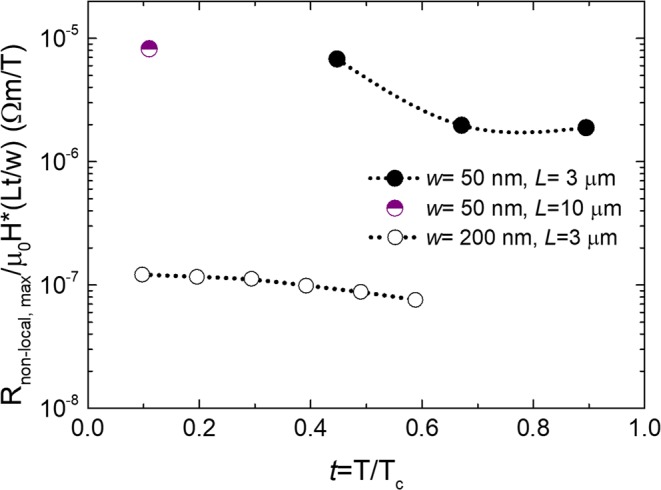
(**a**) Maximum non-local resistance normalized with NWs geometry and magnetic field, as a function of t = T/T_c_.

Using a modified version of Eq. (2), one can compare the non-local electrical signal in WC wires of different dimensions. Figure 4 shows the dependence of R_non-local, max_/μ_0_H × (*Lt*/*W*) with reduced temperature (t = T/T_*c*_). With both NWs having a similar thickness, the signal for 50 nm-wide NWs is nearly two orders of magnitude higher than for the 200 nm-wide one. The measured giant non-local signal in 50 nm-wide NWs confirms our expectations that the vortex row is more rigid than the vortex lattice in wider wires due to its quasi-1D character, and that the higher confinement potential prevents transversal vortex displacements more efficiently. Given that the WC material is the same in both wires, the parameter of vortex viscosity should be taken as an effective one, arising not only from the superconducting material itself but also from the underlying vortex potential and the vortex-vortex interactions.

**Figure 5:**
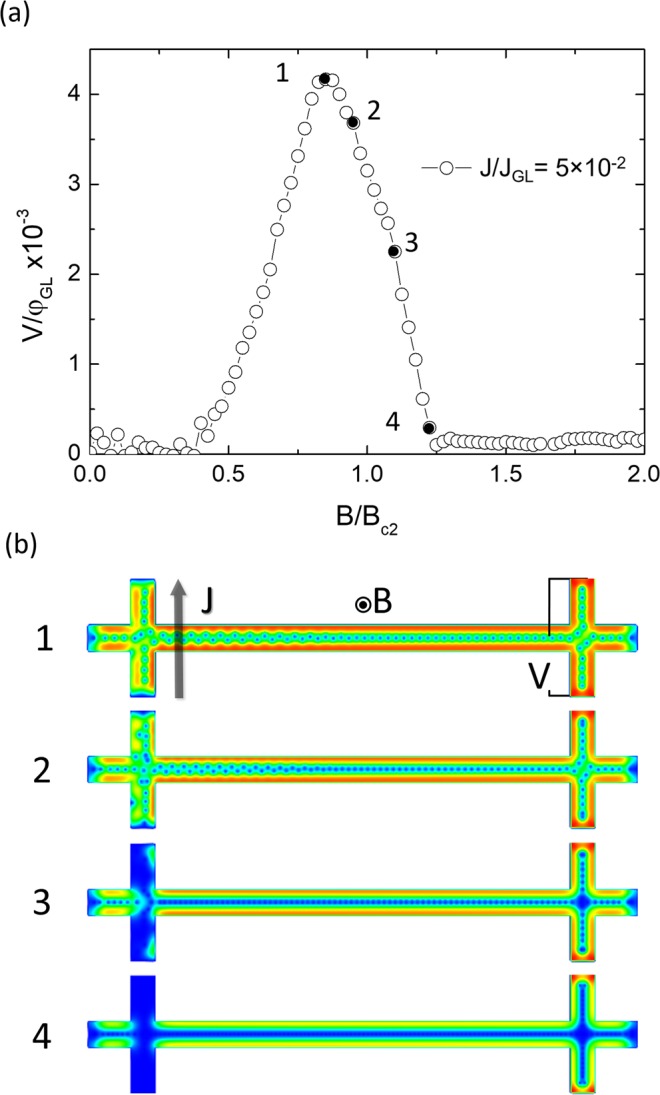
Non-local voltage versus field. (**a**) V(B) obtained from TDGL simulations at current density J = 0.05J_GL_, where interesting features are denoted with black dots (1)–(4). (**b**) Snapshots of the vortex distribution at four points corresponding to (**a**). (1) the maximal voltage, corresponding to the field value for which J = J_c_(B), (2) increased local vortex motion at the current contacts slightly above J_c_(B), (3) state of high dissipation in the current leads, (4) normal-state cut-off point. The vortex density gradient along the path of the NW is accounted for by the decay in momentum transfer that occurs along the longitudinal section of the NW, and by finite size effects in the leads: their presence and the existence of sharp corners create effective barrier walls at the crossing points between the leads and the longitudinal section of the nanowire which distort and compress the lattice.

We performed numerical simulations based on the TDGL framework using a geometry comparable to that of nanostructure A-short (see Experimental section and Supporting information), and its material parameters and experimental conditions. The dependence of the non-local voltage with the applied magnetic field *V*_*non-local*_(B) for a fixed bias current density J = 0.05J_GL_ (see Experimental section) obtained from numerical simulations (Fig. 5(a)) shows an equivalent dependence to that of the experimental data (Figs 2(a) and 3). Snapshots of the vortex distribution in the form of Cooper pair density (CPD) maps at four representative points are shown in Fig. 5(b). Below B~0.4B_c2_, the intrinsic pinning of the material keeps the vortices still, i.e., J < J*(B). At B~0.4B_c2_, the onset of vortex motion occurs at the crossing point between the current lead and the longitudinal section of the nanostructure. Increasing the magnetic field (i.e. the vortex density) within the range 0.4B_c2_ < B < B_c2_ results in an increment of the number of vortices passing through the voltage lead, leading to a higher in non-local voltage signal. Slightly below B_c2_, the vortex density is high enough for J to reach its critical J_c_(B) value, for which vortices at the current lead start to leave it, in particular at the crossing point they move along the longitudinal nanowire. At this stage, the magnitude of the non-local signal decreases as the pressure transferred to the longitudinal section of the nanowire falls down. Further increasing the field eventually drives the whole nanostructure to the normal state, in which no vortices nor driving current flow in the voltage lead, reducing the non-local signal down to zero.

**Figure 6:**
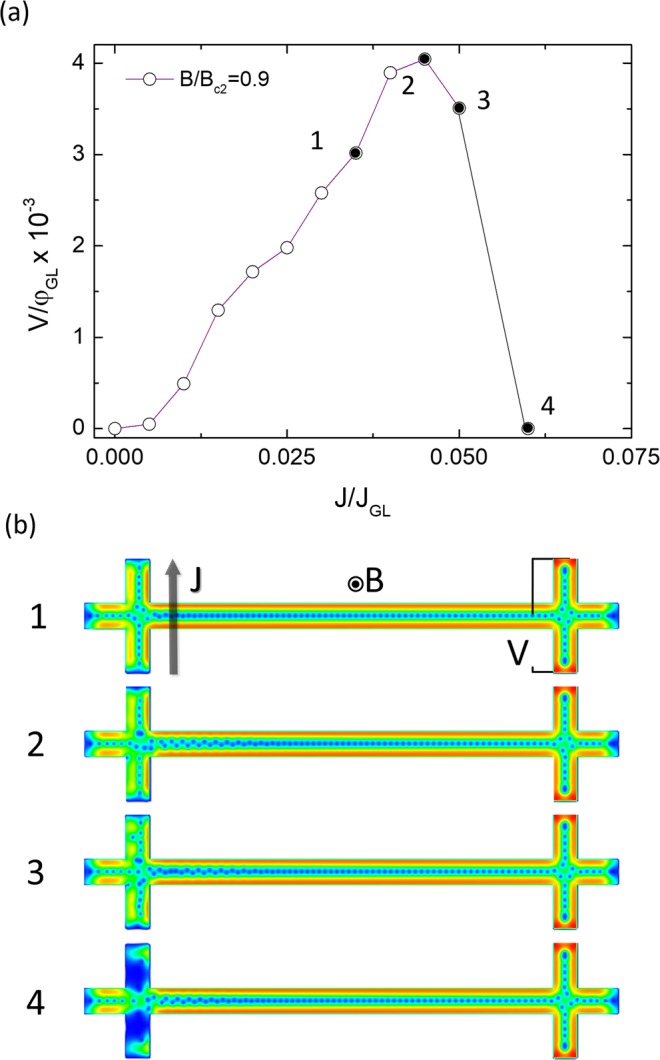
Current-voltage characteristics. (**a**) V(*J*) curves at field B = 0.9B_c2_, obtained from TDGL simulations, where black dots indicate characteristic features of vortex dynamics. (**b**) Snapshots of the vortex dynamics at four points corresponding to (**a**). (1) Regular non-local vortex motion along the nanowire, for J in the range J*(B) < J < J_c_(B) (2) the maximal voltage at J = J_c_(B), (3) state of high dissipation for J > J_c_, (4) snapshot just before completing the transition to the normal state.

Similarly, the dependence of the non-local voltage with the bias current density *V*_*non-local*_(J) for a fixed applied magnetic field B = 0.9B_c2_, (Fig. 6(a)) shows the same dependence to that of the experimental data shown in Fig. 2(b) and inset of Fig. 3. The high vortex density occurring at B = 0.9B_c2_ results in a quick saturation of pinning sites, for which the value of J*(B) is swiftly reached at J ~ 0.006J_GL_. At that point, vortices in the crossing point in the current lead are pushed towards the voltage lead, yielding the non-local voltage as they cross it. Increasing J in the 0.006J_GL_ < J < 0.050J_GL_ range, the magnitude of the non-local signal increases as the pressure exerted at the current lead also grows. J_c_(B) is reached in these conditions at J = 0.050J_GL_, the critical value at which vortices leave the current lead, in particular at the crossing point they move along the longitudinal nanowire. The transition to the normal state at the transversal nanowire reduces the amount of pressure transferred to the longitudinal section of the nanowire, thus decreasing the magnitude of the non-local signal.

## Conclusions

To conclude, we have measured giant non-local electrical signals in 50 nm-wide superconducting WC NWs grown by Focused Ion Beam Induced Deposition. The observed maximum of the non-local resistance is 36 Ω, much larger than in previous works, demonstrating that the single vortex line at the center of these NWs is more rigid due to its quasi-1D-character and their confinement potential. This giant non-local signal is measured after the vortices have travelled 3 μm and 10 μm along a current-free superconducting channel. Remarkably, the R_non-local_ value is 40 times higher than those reported for wider wires of other superconductors^29^. The maximum non-local resistance (normalized by the magnetic field and the dimensions of the NWs) is two orders of magnitude higher for 50 nm-wide NWs than for 200 nm-wide ones. Numerical simulations performed within the TDGL framework support the vortex dynamics interpretation of the experiment and indicate the capability of single-vortex row-hosting nanostructures for long-range vortex transport.

The huge detection of non-local vortex motion along current-free superconducting WC channels of up to 10 μm in length opens a promising path for the development of vortex-based electronics, in which single Abrikosov vortices could be used as quantized information bits for the design of memory cells^40–45^. Since the utilized FIBID technique allows for the fabrication of complex and free-shape structures, one can anticipate the growth of more sophisticated superconducting channels than the present straight NWs, becoming a potential tool for the fabrication of memory cells where the information bit can be written or erased without applying current along the channel. Our results can strengthen this subject for further development of superconducting electronics, renewing challenges for theories and experiments based on the manipulation of single Abrikosov vortices.

## Methods

### Growth of nanowires

We utilize a Ga^+^ FIBID technique in combination with the W(CO)_6_ gaseous precursor material to grow superconducting WC nanostructures with relatively high T_c_~5 K^33^ in a Helios 600 Nanolab (FEI Company) dual beam microscope. In a general way, the precursor flux is delivered into the process chamber, and the Ga^+^ FIB is then scanned over the patterned area inducing the deposition of WC, mainly due to the interaction of secondary electrons with the precursor molecules adsorbed on the substrate surface^32^. The precursor gas was injected into the process chamber using a needle inserted in the vicinity of the specimen, around 50 μm away in *x*/*y* direction and around 150 μm away in *z* direction of the substrate surface, to obtain an efficient FIBID process. The parameters used in the WC FIBID process are: ion beam acceleration voltage = 30 kV and ion beam current~ 1 pA (50 nm NWs) and 29 pA (200 nm NWs); dwell time = 200 ns; scanning pitch = 25.5 nm; chamber base pressure = 1 × 10^−6^ mbar; chamber pressure during FIBID process = 1 × 10^−5^ mbar; precursor temperature = 55 °C. A silicon wafer with a 250 nm thermally grown oxide layer was used as a substrate. A process of optical lithography using lift-off method was performed to fabricate four Ti pads on the substrate with a thickness of 150 nm. Then, four Pt-C FIBID NWs were deposited using (CH_3_)_3_Pt(C_p_CH_3_) precursor material to connect the WC nanostructure to the Ti pads. Studies on W-FIBID films by means of Scanning Tunneling Microscopy and Spectroscopy at very low temperature have shown that this material follows the Bardeen-Cooper-Schrieffer theory and displays a well-defined Abrikosov vortex lattice^39^. In addition, its technological potential has also been demonstrated in applications such as the reparation of damaged micro-SQUIDs, the conversion of SQUIDs into highly sensitive SQUID-susceptometers^46,47^, the fabrication of Josephson junctions^48^ and the creation of three-dimensional nano-SQUIDs^49^.

### Microstructure and composition at the nanoscale

The microstructure and composition of the WC NWs have been studied by Transmission Electron Microscopy (TEM). High resolution TEM (HRTEM), scanning transmission electron microscopy (STEM) imaging and EDS of the NWs were performed in an FEI Tecnai F30 operated at 300 kV. The energy resolution of the EDS experiments was approximately ~125 eV.

### Numerical simulations

The time-dependent Ginzburg-Landau (TDGL) theory allows one to gain insight in the dynamics of vortex-mediated long-range information transfer for superconducting nanowires in the dirty limit^50^. The TDGL method is used to monitor the spatio-temporal evolution of superconducting order parameter ${\rm{\Delta }}({\bf{r}},t)=|{\rm{\Delta }}({\bf{r}},t)|{e}^{i\theta ({\bf{r}},t)}$ ($|{\rm{\Delta }}|$ being the amplitude, and θ the phase of the order parameter):

3 $$\frac{u}{\sqrt{1+{({\rm{\Gamma }}|{\rm{\Delta }}|)}^{2}}}[\frac{\partial }{\partial t}+iV+\frac{\partial }{\partial t}(\frac{{{\rm{\Gamma }}}^{2}{|{\rm{\Delta }}|}^{2}}{2})]{\rm{\Delta }}\,=[{(\nabla -i{\boldsymbol{Q}})}^{2}+(1-{|{\rm{\Delta }}|}^{2})]{\rm{\Delta }}$$

Equation (3) is solved self-consistently with the equation for the electrostatic potential V(**r**,t):

4 $${\nabla }^{2}V=\nabla (Im\{{\rm{\Delta }}\ast [\nabla -i{\boldsymbol{Q}}]{\rm{\Delta }}\})$$

Equations (3) and (4) are given in the dimensionless form. The distances are expressed in units of the coherence length at the temperature T, ξ(T), the time is in units of ${\tau }_{GL}(T)=\pi \hslash /8{k}_{B}{T}_{c}uf(T),$ where *u* = 5.79 is the ratio of the relaxation times for the order parameter amplitude and phase, in the dirty limit^50^. The complex order parameter Δ is given in units of ${{\rm{\Delta }}}_{GL}(T)=4\sqrt{u}{k}_{B}{T}_{c}\sqrt{f(T)}/\pi \sqrt{g(T)}$ and the electrostatic potential V is in units of ${\phi }_{GL}(T)=\hslash /{e}^{\ast }{\tau }_{GL}(T)$. The vector potential **Q** is given in the ▽**Q** = 0 gauge, with units of ${\phi }_{GL}$/2πξ(T), and the current density J is given in units of J_GL_(T) = *σ*_*n*_φ(T)g(T)/ξ(T), where *σ*_*n*_ is the normal-state conductivity. The parameter ${\rm{\Gamma }}=2{{\rm{\tau }}}_{{\rm{in}}}{{\rm{\Delta }}}_{GL}(T)/\hslash$ contains the influence of the inelastic phonon-electron scattering time ${{\rm{\tau }}}_{{\rm{in}}}$ on the dynamics of the superconducting condensate. The functions *f*(T) = (1 − T^2^/T_c_^2^) (1 + T^2^/T_c_^2^)^−1^ and *g*(T) = (1 + T^2^/T_c_^2^)^−2^ are thermal kernels used to describe the behavior of the superconducting condensate far away from the critical temperature T_c_.

The simulations were implemented using a finite difference method, on a Cartesian map with a dense grid spacing of 0.2 ξ, where a simplified version of the geometry of the experimental nanostructure A-short was reproduced. Specifically, it had a width of 50 nm, length of 1160 nm, and two 50 nm-wide and 240 nm-long transversal contacts.

No current density flows through the superconductor-vacuum (SI) boundary: **n**_*SI*_(▽ - i**Q**)Δ = 0 and **n**_*SI*_▽V = 0 (**n**_*SI*_ is unit vector perpendicular to the SI boundary); while at the superconductor-normal metal (SN) boundary, where the external current J is injected and fully transformed into the normal current component: **n**_*SN*_▽V = ± J, with vanishing order parameter Δ|_SN_ = 0 (**n**_*SN*_ is the unit vector perpendicular to the SN boundary region).

To account for the smooth decay in thickness occurring at the endings of the real nanostructure due to precursor-limited growth regime, the edge barriers for vortex entry and exit were weakened at these spots by modifying the functions *f*(T) and *g*(T) (“open boundary conditions”).

## Supplementary information

Long-range vortex transfer in superconducting nanowires
