# Supplementary material for: Long-range vortex transfer in superconducting nanowires
Source: Sci Rep. 2019 Aug 27;9:12386. doi: 10.1038/s41598-019-48887-7 (PMC6712003; doi:10.1038/s41598-019-48887-7)
Supplement: Supplementary file 1 — Long-range vortex transfer in superconducting nanowires [file 41598_2019_48887_MOESM1_ESM.docx]

Supporting Information for

Long-range vortex transfer in superconducting nanowires

Rosa Córdoba^1,2*^, Pablo Orús^1,2^, Željko L. Jelić^3^, Javier Sesé^1,2,4^, Manuel Ricardo Ibarra^1,2,4^, Isabel Guillamón^5^, Sebastián Vieira^5^, Juan José Palacios^6^, Hermann Suderow^5^, Milorad V. Milosević^3^ and José María De Teresa^1,2,4*^

^1^ Instituto de Ciencia de Materiales de Aragón (ICMA), Universidad de Zaragoza-CSIC, E-50009 Zaragoza, Spain

^2^ Departamento de Física de la Materia Condensada, Universidad de Zaragoza, E-50009 Zaragoza, Spain

^3^ University of Antwerp, Department Physics, Groenenborgerlaan 171, B-2020 Antwerp, Belgium.

^4^ Laboratorio de Microscopías Avanzadas (LMA)-Instituto de Nanociencia de Aragón (INA), Universidad de Zaragoza, E-50018 Zaragoza, Spain

^5^ Laboratorio de Bajas Temperaturas, Departamento de Física de la Materia Condensada, Instituto de Ciencia de Materiales Nicolás Cabrera, Condensed Matter Physics Center (IFIMAC), Universidad Autónoma de Madrid, 28049, Madrid, Spain

^6^ Departamento de Física de la Materia Condensada, Condensed Matter Physics Center (IFIMAC), Universidad Autónoma de Madrid, 28049, Madrid, Spain

^7^ Present address: Instituto de Ciencia Molecular, Universitat de València, Catedrático José Beltrán 2, 46980 Paterna, Spain

**Microstructure and composition at the nanoscale**

Figure S1 shows a typical HRTEM image of a cross-sectional view of a WC nanowire of 50 nm in width. The grain size is very small, which makes the indexation of the weak spots observed in Fast Fourier Transforms (FFTs) unfeasible, and thus does not allow to properly identify the crystallographic structure. Note that it is exceptionally complicated to study this type of samples due to the high atomic number of tungsten and the very small grain size. By carefully analyzing FFTs of squared areas in the HRTEM image, we can identify a few weak diffraction spots, indicating the existence of short-range crystallographic order. Scanning Transmission Electron Microscopy-Energy Dispersive X-ray Spectroscopy (STEM-EDS) experiments indicate that the composition of the WC nanowire is almost homogeneous along the nanowire width and thickness. The atomic percentage of each element is W= 40 % ± 7 %, C= 43 % ± 4 %, Ga= 10 % ± 3 %, O= 7 % ± 2 %.

|  |
| --- |
| **Figure S1.** HRTEM image of a cross-sectional view of the 50 nm-wide WC nanostructure. |

**Local electrical signal in WC nanowires of 50 nm in width**

Magnetotransport properties were studied in a commercial Physical Property Measurement System (PPMS) from Quantum Design. The temperature range was varied from 300 K down to 2 K, and magnetic fields between ±9 T were applied perpendicular to the substrate.

|  |
| --- |
| Figure S2: Electrical measurements of the WC nanostructure in the local configuration. (a) Normalized resistance as a function of temperature. Inset shows an SEM image of the WC nanostructure of 50 nm in width. (b) Normalized resistance as a function of applied magnetic field at different temperatures. |

Figure S2(a) shows the normalized resistance (R/R_N_) as a function of temperature for the 50 nm-wide nanowires. The resistance at the normal state (10 K) R_N_ is 6563 Ω and its resistivity, ρ_N_= 219 μΩcm, are in agreement with previous values reported in the literature ^1,2^. At low temperature, the nanowire shows a sharp resistance drop at T_c_≈ 4.47 K (defined as the temperature at which the resistance value is 0.5R_N_), entering the superconducting state.

Figure S2(b) shows the resistance versus applied magnetic field of this nanowire at various temperatures (0.76T_c_ - 0.83T_c_). A reentrance of the superconductivity induced by high magnetic fields ~1.85 T is observed in these curves, as previously reported for this material ^2^.

**Non-local electrical signal in WC nanowires of 200 nm in width**

Figure S3 shows SEM images of the experimental configuration for the non-local electrical measurements in 200 nm-wide WC nanowires.

|  |
| --- |
| Figure S3: (a) SEM image of the experimental non-local configuration for electrical measurements of the 200 nm-wide WC FIBID nanostructure. (b) High magnification SEM image of the WC FIBID nanostructure squared area in (a). |

**Dependence of the non-local signal as a function of applied magnetic field and the bias current for WC nanowires of 200 nm in width**

The R_non-local_ was studied in 200 nm-wide WC NWs applying magnetic fields up to ±9 T perpendicularly to the substrate, and in temperatures from 0.5 to 5 K ^3^. By fixing the values of temperature and current, the non-local signal is observed starting from a certain magnetic field. This field value can be related to the vortex lattice depinning, which is normally associated to the saturation of pinning centers of the interstitial vortices at high vortex densities ^4^. Figure S4(a) shows R_non-local_ and R_local_ as a function of the magnetic field at 2 K and 0.5 μA. The dependence of the R_non-local_ is symmetrical at positive and negative magnetic fields.

|  |
| --- |
| Figure S4. (a) For the 200 nm-wide NW, magnetic field dependence of the non-local resistance (left *y*-axis) and local resistance from the local geometry measurement (right *y*-axis), at 2 K (0.4T_c_) and 0.5 μA bias current. (b) Bias current dependence of the non-local voltage at 2 K (0.4T_c_) and at -5 T magnetic field using DC measurement mode, and at 5 T using AC measurement mode. |

Figure S4(b) shows the V_non-local_ as a function of the current measured in DC mode from 20 μA to -20 μA at 2 K and -5 T together with the V_non-local_ from 0 to 20 μA at 2 K and 5 T measured in AC mode. First, we highlight that the non-local voltage changes sign under negative current and shows a linear dependence from 5 μA to -5 μA, thus having the same dependence as the measurement obtained in the AC mode. By increasing the applied current around the critical current, the non-local voltage now has the same sign for positive and negative current, whereas it vanishes in the AC mode. We ascribe this behavior to thermal effects: in the DC mode, a temperature gradient can be produced in the nanostructure, causing a spurious non-local signal.

From R_non-local_ as a function of the magnetic field curves at several temperatures, from 0.5 to 4 K and 1 μA of an applied current, we extract the values of the maximum R_non-local_ and the magnetic field values at which the R_non-local_ is detected, B_min_, B (R_non-local, max_) and B_max_ (as indicated in Figure S4(a)). Figure S5(a) shows the maximum R_non-local_/μ_0_H as a function of temperature t=T/T_c_ from 0.1 to 0.6. (0.042 ΩT^-1^ at 0.5 K). Figure S5(b) depicts the temperature and magnetic field range at which the non-local signal is detected.

|  |
| --- |
| Figure S5. (a) For the 200 nm-wide NW, temperature dependence of the normalized maximum non-local resistance. (b) Temperature dependence of the magnetic field range in which non-local resistance has been detected. |

**Numerical simulations performed within the time-dependent Ginzburg-Landau (TDGL) equations**

When performed in the precursor-limited regime, the areas located at the geometrical ends of nanostructures grown by FIBID present a smooth decay in thickness rather than abrupt, sharp edges, such an effect was accounted for in the numerical simulations by gradually lowering the barrier walls at the ends of the longitudinal part of the NW (*open boundary conditions)*.

In addition to reproducing experimental data, the tool was utilized to explore the potential range of long-range vortex transport in these nanostructures, with the results being summarized in figure S6. In a simulated geometry equivalent to that of nanostructure A-short, the initial positions of vortices were recorded for both open and closed boundary conditions (BCs) (Figure S6(a)) after fixing the value of the magnetic field. A similar trend in the inter-vortex separation was found in both types BCs.

Then a long, finite driving current pulse was applied at the current lead. The relative displacement of each individual vortex as a function of the distance to the current injection point after this pulse was applied (*vortex* *transfer length),* was then recorded. In open BCs, the vortex displacement (i.e. momentum transfer) gradually decayed as the distance to the current lead increased, tending towards finite values of momentum transfer at high values of transfer length (Figure S6(b)). In closed BCs a much more pronounced decay was observed, with the momentum transfer going to zero with increasing transfer length. (Figure S6(c)). Moreover, the vortex displacement fell below the onset threshold (corresponding a displacement of one inter-vortex distance) in most of the simulated magnetic field values.

Thus, provided that the two ends of the nanowire behave as open BCs, long-range vortex transport is not only enhanced, but sustained in long range, when a single row of vortices is hosted within the nanostructure.

|  |
| --- |
| Figure S6. (a) Average vortex-vortex separation [*a*_v−v_] (in units of the coherence length ξ) as a function of the applied magnetic field (scaled with the upper critical field B_c2_), for open (blue open circles) and closed (black full squares) boundary conditions (BC). (b-c) Propagation of non-local displacement of vortices along the nanowire (b) for open BC (c) for closed BC at different magnetic fields, in units of inter-vortex separation a_v-v_. After applying a long, finite driving current pulse at each value of magnetic field (step: 0.05B_c2_), the individual displacement of each vortex is recorded, relative to its starting equilibrium position. The dashed line denotes the threshold for vortex transport, corresponding to a displacement equal to one inter-vortex separation. |

1. Sadki, E. S., Ooi, S. & Hirata, K. Focused ion beam induced deposition of superconducting thin films. *Phys. C-Superconductivity Its Appl.* **426**, 1547–1551 (2005).

2. Córdoba, R. *et al.* Magnetic field-induced dissipation-free state in superconducting nanostructures. *Nat. Commun.* **4**, 1437 (2013).

3. Córdoba Castillo, R. *Functional Nanostructures Fabricated by Focused Electron/Ion Beam Induced Deposition*. *Springer Theses* (Springer International Publishing, 2014). doi:10.1007/978-3-319-02081-5

4. Helzel, A. *et al.* Nonlocal vortex motion in mesoscopic amorphous Nb_0.7_Ge_0.3_structures. *Phys. Rev. B* **74**, 220510 (2006).
